# Observations upon Primary and Secondary Amputation

**Published:** 1854-09

**Authors:** W. Stone

**Affiliations:** New Orleans


### SELECTIONS.

From the New Orleans Medical News.

*Observations upon Primary and Secondary Amputation.* By Prof. W. STONE, M.D., of New Orleans.

THE principle of immediate amputation, although beyond all doubt correct, has caused the loss of countless limbs unnecessarily, and, I believe, of as many lives as it has saved. The error, evidently, is from over-estimating the security afforded by primary over secondary amputation. The first duty of the surgeon certainly is to secure, if possible, the life of his patient; and the second, to preserve as much of his person in as perfect a manner as possible. In the anxiety to fulfil the first duty, by over-estimating the security which amputation affords, limbs are often sacrificed that are curable, and by disregarding the proper time for amputation, a life may be lost that would have been safe without an operation. In severe injuries of the extremities, if fatal, death is produced either by the concussion or subsequent pain and suppuration which exhausts the patient; or it may occasionally be from tetans or gangrene. Against the first cause of death amputation affords no security—on the contrary, it favors it. The question of amputation before reaction, I believe, is settled by every American surgeon of experience in the negative. This subject was sharply discussed in England on the occasion of the death of the celebrated statesman, Huskisson, who had both legs or thighs crushed on the Liverpool and Manchester Railroad. The Liverpool surgeons attempted to bring on reaction, but every means failed; the concussion had thrown him into a fatal collapse. The London surgeons took the matter up, blamed the Liverpool surgeons, and urged that immediate amputation should have been resorted to, and talked nonsensically of the stimulus of the knife. When one hears such reasoning, he feels the truth of the remark made by some one in the last century, that surgeons were bad pathologists, and worse physiologists.

In severe injuries, where the patient is thrown into collapse, and amputation is necessary or unavoidable, if the case is critical, it is a nice point to decide when, exactly, it can be performed with the most safety. If the patient were in great agony, and amputation could relieve it, there could be no doubt of the propriety of amputating at once, no matter what the state of pulse might be; but this is not the case; the shock has been received, the mischief has been done, the parts are in a measure paralyzed, and no very severe pain takes place until reaction. The question in such cases is, whether the injured limb is a greater source of pain than the stump would be after amputation; and considerable allowance should be made for the shock of the operation. The discovery of chloroform enables us, in a great measure, to avoid the shock of the stimulus of the knife, but not entirely. My experience is, that when amputation is unavoidable, it is best to do it as soon as reaction has fairly commenced, while the patient is under the influence of the first shock of the injury, the pulse flickering, etc. Any disturbance of the system, pain, or loss of blood, might cause a fatal collapse in a case that would be perfectly safe if managed with tact and judgment. By reaction I do not mean a full resistant pulse. The nervous system receives the shock, and is the first to react, as shown by the increased sensibility and improved capillary circulation, before any perceptible improvement in the pulse is observed. This, however, soon follows, and the pulse becomes more steady. When the system is suffering from a severe injury, it is often the case that stimulants do not act as such when put upon the stomach. In extreme cases, when the patient is in danger of collapse, it is evident to me that the stomach does not absorb, but is nauseated, and all the pressing effects of nausea are produced. The rectum can scarcely be said to sympathize with the system in general, and always preserves an active absorbing surface. Stimulants given by injection produce ready effect; and I always use my stimulants in this way where the patient is in danger, even when he is perfectly able to swallow, for they are much more prompt and effective. If too long a time elapses after an injury before amputation, the sensibility of the limb, which was at first paralyzed, becomes highly exalted; and although we can, by the use of chloroform, prevent the shock from the operation, we have fresh wounds in parts in a morbid state; the stump is much more painful, and, as a general rule, does not do as well as when the operation is performed earlier. By the above I mean a state of the parts before any decided inflammatory action has taken place, and my firm conviction is, that where no large joints are involved, or parts injured that will give extreme pain to the patient, he will have a better chance for his life if we give him a chance for his limb also, even if we have to resort to secondary amputation—I mean if the most favorable period for operating has passed.

